# TH Open Continues to Highlight the State-of-the-Art on Thrombosis and Hemostasis with a Renewed Editorial Board

**DOI:** 10.1055/s-0044-1785515

**Published:** 2024-04-08

**Authors:** Rory R. Koenen

**Affiliations:** 1 Department of Biochemistry, CARIM School for Cardiovascular Diseases, Maastricht University, Maastricht, The Netherlands

*TH Open* is the open access companion journal to *Thrombosis and Haemostasis* founded in 2017 and publishes basic and clinical scientific studies on all aspects of hemostasis and thrombosis.

My name is Rory Koenen. I am a professor of Biochemistry of Vascular Inflammation and Thrombosis at Maastricht University. My research interests are the intersection of thrombosis and inflammation with a focus on platelets, extracellular vesicles, and the actions of coagulation factors during inflammation. I took over the position of the editor-in-chief of *TH Open* in January 2024. I wish to thank all (past) members of the *TH Open* editorial board, the editors-in-chief of *Thrombosis and Haemostasis* , Christian Weber and Gregory Lip, as well as the publisher Thieme Group for their confidence and support. I look forward to our collaboration.

The year 2024 starts with a renewed editorial board hyperlink ( https://lp.thieme.de/open-access-files/209/editorial_board.pdf ) to reflect diversity in expertise, geographical location, and gender to cover the various aspects of an ever-expanding scientific field. The field of hemostasis and thrombosis continues to be exciting, with its many aspects ranging from novel therapeutics for the treatment of hemophilia to new drugs to prevent arterial and venous thrombosis. 1 2 3 The management of both bleeding and thrombosis continues to raise relevant questions of clinical and practical nature, and must also draw novel insights from fundamental science. In particular, the recent COVID-19 pandemic has emphasized that basic science is fundamental in understanding the thrombotic complications driven by this novel viral infection and has highlighted the interplay between the immune system and hemostasis. 4 5 Lessons from the mechanisms underlying COVID-19-associated thrombosis might lead to the discovery of interesting drug targets for the prevention and treatment of thrombosis in general. This is highly desirable, as thrombosis is still the number one cause of death in Western countries, 6 and the causes of many thrombotic disorders, such as cancer-related thrombosis, autoimmune thrombosis, and more recently vaccine-induced thrombosis are still largely unknown. Associated with this is the lack of causative therapeutic options for these thrombotic disorders. On the other side of the spectrum is bleeding, as a complication of cancer, an adverse effect of antithrombotic therapy or due to hemophilia. Although advances have been made in the management of bleeding in hemophilia and in increasing the safety of antithrombotic drugs, challenges are still present, for example, in hemophiliacs requiring surgery 7 and in elderly patients receiving anticoagulation during atrial fibrillation. 8

For these and many other questions in the field of hemostasis and thrombosis, *TH Open* continues to be a state-of-the-art open access platform. *TH Open* welcomes first submissions of original relevant work, as well as previously submitted quality work from *Thrombosis and Haemostasis* and other specialty journals in the field that did not receive sufficient priority. Quality and integrity of research is important to all of us. Therefore, all submissions will undergo an independent peer review and will be handled with appropriate care, speed, and consideration.

On behalf of the editorial board, I look forward to receiving your exciting work for consideration and to bring the state of the art on hemostasis and thrombosis to you.

With best wishes,

Rory R. Koenen, PhD

Editor-in-chief
